# A preponderance of gastrointestinal cancer patients transition into cachexia syndrome

**DOI:** 10.1002/jcsm.13086

**Published:** 2022-09-27

**Authors:** Linda Anne Gilmore, Santiago Olaechea, Brian W. Gilmore, Bhavani S. Gannavarapu, Christian M. Alvarez, Chul Ahn, Puneeth Iyengar, Rodney E. Infante

**Affiliations:** ^1^ Department of Clinical Nutrition University of Texas (UT) Southwestern Medical Center Dallas TX USA; ^2^ Center for Human Nutrition University of Texas (UT) Southwestern Medical Center Dallas TX USA; ^3^ Department of Computer Science and Engineering University of North Texas Denton TX USA; ^4^ Department of Radiation Oncology University of Texas (UT) Southwestern Medical Center Dallas TX USA; ^5^ Harold C. Simmons Comprehensive Cancer Center University of Texas (UT) Southwestern Medical Center Dallas TX USA; ^6^ Department of Internal Medicine University of Texas (UT) Southwestern Medical Center Dallas TX USA

**Keywords:** colorectal cancer, gastroesophageal cancer, hepatobiliary cancer, pancreatic cancer, weight loss

## Abstract

**Background:**

Cancer cachexia is frequently documented by self‐reported, single time‐point weight histories. This approach lacks the granularity needed to fully elucidate the progression of cachexia syndrome. This study aimed to longitudinally assess body weight changes pre‐ and post‐cancer diagnosis in gastrointestinal (GI) cancer patients.

**Methods:**

Body weights and relevant clinical data recorded in the electronic health record 12 months pre‐ and post‐GI cancer (colorectal, gastroesophageal, hepatobiliary and pancreatic) diagnosis were extracted. Weight loss was categorized by the International Consensus Definition for cachexia.

**Results:**

A total of 879 patients were included in the final cohort including patients diagnosed with colorectal (*n* = 317), hepatocellular (*n* = 185), biliary (*n* = 72), pancreatic (*n* = 186) or gastroesophageal (*n* = 119) cancer. Stage of disease was equally distributed. Patients without cachexia at diagnosis (*n* = 608) remained weight stable during the 12 months pre‐diagnosis (+0.5 ± 0.5% body weight; *P* = 0.99). Patients with cachexia at diagnosis (*n* = 271) remained weight stable 6 to 12 months prior to diagnosis (+0.4 ± 0.8%; *P* > 0.9999) and lost 8.7 ± 0.6% (*P* < 0.0001) within the 6 months pre‐diagnosis. Patients without cachexia at diagnosis lost more weight post‐diagnosis (6.3 ± 0.6%) than patients with cachexia at diagnosis (4.7 ± 1.0%; *P* = 0.01). Pre‐diagnosis weight trajectories did not differ between primary malignancies or stage of disease in patients without or with cachexia at diagnosis (all *P* ≥ 0.05). Post‐diagnosis weight trajectories did differ by primary malignancy (*P* ≤ 0.0002) and stage (*P* < 0.0001). In both patients without and with cachexia at diagnosis, colorectal patients lost the least amount of weight post‐diagnosis and gastroesophageal patients lost the most amount of weight post‐diagnosis. Stage 4 patients without or with cachexia at diagnosis lost the most weight post‐diagnosis (*P* ≤ 0.0003). Regardless of cachexia status at diagnosis, patients lost more weight when treated with systemic therapy (7.1 ± 0.7%; *P* < 0.0001; *n* = 419) or radiation therapy (8.4 ± 1.4%; *P* = 0.02; *n* = 116) compared to those who did not. Patients who did not have surgery lost more weight post‐diagnosis (7.6 ± 1.1%; *P* < 0.0001; *n* = 355) compared to those who did have surgery. By 12 months post‐diagnosis, 83% of the surviving GI cancer patients in this cohort had transitioned into cachexia syndrome.

**Conclusions:**

Significant weight loss in patients with GI cancer cachexia at diagnosis initiates at least 6 months prior to diagnosis, and most patients will transition into cachexia syndrome post‐diagnosis, regardless of pre‐diagnosis weight change and stage of disease. These findings punctuate the importance of weight surveillance in cancer detection and earlier palliative interventions post‐diagnosis in the GI cancer patient population.

## Introduction

Cancer cachexia is a progressive, multifactorial syndrome associated with metabolic alterations and systemic inflammation leading to anorexia and loss of fat and lean mass.^1^, ^2^ As cachexia progresses, it becomes less responsive to pharmacological and nutritional interventions and has significant impact on patient morbidity and mortality.^3^, ^4^, ^5^ The prevalence of cachexia at diagnosis, defined by weight loss of ≥5% over a 6‐month time frame as per the International Consensus Definition (ICD),^6^ ranges from 40% to 80% in patients with gastrointestinal (GI) cancers.^5^ Cachexia is thought to be responsible for 30% of all cancer‐related deaths and associated with reduced survival independent of other clinical factors.^7^, ^8^ There are currently no durable treatments for cachexia, though early palliative care interventions may offer survival and quality of life benefits.^5^, ^9^

Cancer cachexia is most commonly diagnosed retrospectively at initial presentation for cancer via self‐reported weight loss by the patient over the preceding 6 months using single time‐point weight histories.^10^ The rate of weight loss pre‐ and post‐cancer diagnosis remains unclear. This lack of granularity makes it difficult to fully appreciate the progression of cancer cachexia's physical manifestations and how cancer cachexia evolves with the development and progression of cancer. Likewise, the prevalence and progression of cachexia after a cancer diagnosis, as well as the impact of tumour‐directed treatment and/or disease progression, are difficult to ascertain. A comprehensive understanding of pre‐diagnosis body weight trajectories may be critical for earlier cancer detection and cachexia interventions.^11^ In parallel, characterizing the body weight trajectories post‐diagnosis can define the treatment‐related weight loss component of cachexia.

Here, we present a retrospective cohort of 879 patients diagnosed with GI cancers (colorectal, gastroesophageal, pancreatic or hepatobiliary) in which body weight was measured in the clinic at 3‐month intervals 12 months before and after cancer diagnosis. Detailed longitudinal body weights pre‐ and post‐diagnosis revealed that (1) GI cancer patients with cachexia at diagnosis lost their weight within 6 months prior to diagnosis and (2) 95% of surviving GI cancer patients eventually have weight loss with 83% of patients reaching the ICD consensus definition of cachexia syndrome. These findings highlight the need to evaluate weight loss in the general population with greater scrutiny for earlier cancer detection that would facilitate improved survival and quality of life. Additionally, our data indicate that ongoing assessment for weight changes during cancer treatment is warranted to identify those patients that transition into cachexia syndrome post‐cancer diagnosis allowing for earlier palliative care interventions.

## Methods

### Population cohort and data acquisition

This study was approved by the University of Texas (UT) Southwestern Institutional Review Board (IRB) prior to data acquisition (Protocol STU 092013‐028). Data were automatically and manually extracted from the electronic health record (EHR) and institutional tumour registry. Patients included in the cohort were diagnosed with a GI cancer including colorectal, gastroesophageal, hepatic, biliary or pancreatic cancer at UT Southwestern Medical Center from May 2005 to December 2019. Retrospective inpatient and outpatient clinical data were extracted including patient characteristics (i.e., sex, race and age at cancer diagnosis), anthropometrics including height and body weights, cancer diagnosis date, tumour characteristics from pathology reports, cancer‐related treatment data and current vital status.

### Organization of body weight data

All body weights recorded in the patient's EHR pre‐ and post‐cancer diagnosis were extracted.

Excel macros were written to longitudinally organize body weights in 3‐month intervals 1 year pre‐ and post‐cancer diagnosis. The body weight assigned to each 3‐month time point was the body weight closest to that time point within ±45 days. In the instance there was more than one body weight measurement for a specific 3‐month interval, the body weight that was closest to the 3‐month time point was chosen. Although a 45‐day window was allowed, the selected weights were 16.9 ± 12.8, 8.2 ± 8.3 and 11.0 ± 10.8 days from the time point of interest pre‐diagnosis, diagnosis and post‐diagnosis, respectively. Patients included in the final cohort were required to have a body weight recorded at diagnosis and at least one body weight pre‐cancer diagnosis. Due to inherent differences in absolute body weight between patients and patient characteristics, per cent change relative to diagnosis weight was calculated, unless otherwise indicated. Body mass index (BMI) was calculated and classified as underweight, normal weight, overweight or obese as per the World Health Organization criteria.^12^

### Cancer‐associated weight loss

Cancer‐associated weight loss was based on the ICD of cachexia summarized as ≥5% weight loss for patients with BMI ≥ 20 kg/m^2^ or unintentional weight loss ≥2% for patients with a BMI < 20 kg/m^2^.^6^ To evaluate if patients transitioned through the stages of cachexia—no cachexia, pre‐cachexia, cachexia and death—and how many days they remained in each stage of cachexia, patients in our cohort were categorized as having no cachexia (no weight loss or having weight gain), pre‐cachexia (1–4.9% weight loss if BMI ≥ 20 kg/m^2^ or 1–1.9% weight loss if BMI < 20 kg/m^2^), cachexia (≥5% if BMI ≥ 20 kg/m^2^ or ≥2% weight loss if BMI < 20 kg/m^2^) or death if the patient died prior to the time point of interest. Once a patient was categorized with cachexia, they were not categorized as pre‐cachexia or no cachexia. In order to designate a cachexia stage for patients at every time point, at least 6 months of weight measurements prior to each time point was required. Therefore, the classification began at 6 months prior to diagnosis. Time in each stage of cachexia was determined using all available body weights in the EHR.

### Statistical analysis

Descriptive statistics were used to summarize patient and tumour characteristics. Student's *t* test was used to compare baseline characteristics between cachexia status groups for continuous variables. Chi‐squared or Fisher's exact tests were used to test associations for categorical variables and Mann–Whitney *U* test for ordinal variables between cachexia and non‐cachexia groups. Repeated measures mixed‐effect model using the restricted maximum likelihood method was used to compare weight change over time between groups. A significance level was adjusted for multiple comparisons using Tukey adjustment. All tests were two‐sided and performed at 5% significance level. Statistical analyses were performed using GraphPad Prism Version 9.1.0 (San Diego, California).

## Results

**Figure 1:**
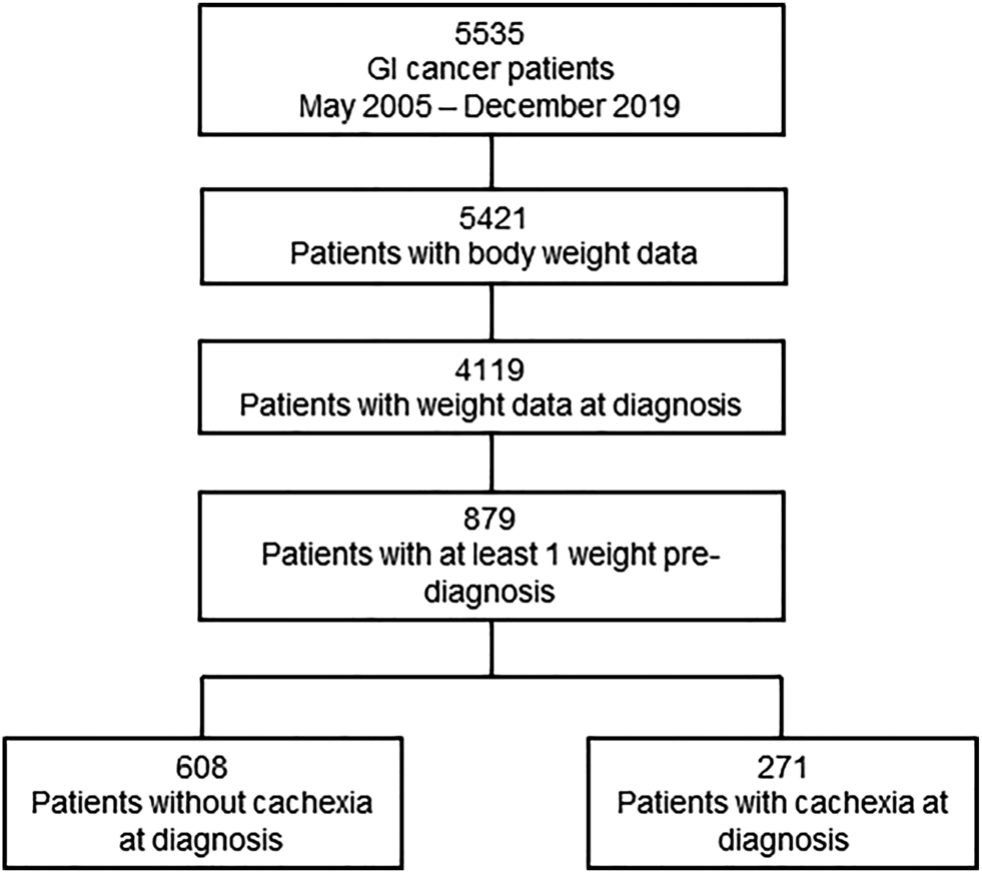
Consort diagram of patients included in the study. Gastrointestinal (GI) cancers include gastroesophageal, colorectal, hepatobiliary and pancreatic cancers.

Through a UT Southwestern Medical Center IRB‐approved protocol, we identified 5535 patients who were diagnosed with a primary GI cancer between May 2005 and December 2019. Patients that did not have a weight measurement at diagnosis and at least one prior to diagnosis were excluded, resulting in a final analysable cohort of 879 patients (*Figure* *1*). In the final cohort, 317 patients (36%) were diagnosed with colorectal cancer, 185 patients (21%) with hepatocellular cancer, 72 patients (8%) with biliary cancer (cholangiocarcinoma, gallbladder or mixed cholangiocarcinoma/hepatocellular), 186 patients (21%) with pancreatic cancer and 119 patients (14%) with gastroesophageal cancer (*Table* *1*). Stage of disease was equally distributed across the cohort except for a higher number of patients with Stage 1 cancers (34%), and a majority of the patients were treated with surgery (60%) and/or chemotherapy (48%) as a part of their therapeutic plan. Overall, the patient cohort had the expected frequencies of White, Black and Asian populations representative of the catchment area.

**Table 1:** Patient characteristics

	Total cohort (*n* = 879)	No cachexia (*n* = 608)	Cachexia (*n* = 271)	*P*‐value
Primary malignancy^a^				0.002
Gastroesophageal	119 (14%)	85 (71%)	34 (29%)	
Colorectal	317 (36%)	229 (72)	88 (28%)	
Hepatocellular	185 (21%)	139 (75%)	46 (25%)	
Biliary	72 (8%)	48 (67%)	24 (33%)	
Pancreatic	186 (21%)	107 (58%)	79 (42%)	
Stage				0.10
1	299 (34%)	227 (76%)	72 (24%)	
2	200 (23%)	141 (71%)	59 (30%)	
3	158 (18%)	114 (72%)	44 (28%)	
4	209 (24%)	118 (56%)	91 (44%)	
Unknown	13 (1%)	8 (62%)	5 (38%)	
Treatment				
Any treatment	773 (88%)	546 (71%)	227 (29%)	0.53
No treatment	106 (12%)	62 (58%)	44 (42%)	
Surgery	524 (60%)	381 (73%)	143 (27%)	0.007
No surgery	355 (40%)	227 (64%)	128 (36%)	
Chemotherapy	419 (48%)	287 (69%)	132 (31%)	0.71
No chemotherapy	460 (52%)	321 (70%)	139 (30%)	
Radiation	116 (13%)	85 (73%)	31 (27%)	0.33
No radiation	763 (87%)	523 (69%)	240 (31%)	
Immunotherapy or biological therapy	19 (2%)	11 (60%)	8 (40%)	0.32
No immunotherapy or biological therapy	860 (98%)	597 (69%)	263 (31%)	
BMI at diagnosis (kg/m^2^)^b^	28.0 ± 6.4	29.2 ± 6.3	25.4 ± 5.7	<0.0001
BMI class at diagnosis^c^				0.34
Underweight	29 (3%)	8 (28%)	21 (72%)	
Normal weight	257 (29%)	139 (54%)	118 (46%)	
Overweight	310 (35%)	227 (73%)	83 (27%)	
Obese	283 (32%)	234 (83%)	49 (17%)	
Age (year)	66.4 ± 12.0	66.1 ± 11.9	67.1 ± 12.0	0.23
Sex				0.51
Male	495 (56%)	347 (70%)	148 (30%)	
Female	384 (44%)	261 (68%)	123 (32%)	
Race				0.56
White	682 (78%)	466 (68%)	216 (32%)	
Black	154 (18%)	112 (73%)	42 (27%)	
Asian/Other	43 (5%)	30 (70%)	13 (30%)	

Abbreviation: BMI, body mass index.

^a^ Categorical variables are frequency (% within column for total cohort and within row for cachexia status). *P*‐value is from *χ* ^2^ or Fisher's exact test for categorical variables and Mann–Whitney *U* for ordinal variables between cachexia status groups.

^b^ Continuous variables are mean ± SD. *P*‐value is from a two‐sided Student’s t‐test between cachexia status groups.

^c^ Body mass index (BMI) classification based on World Health Organization categories: underweight <18.5 kg/m^2^, normal weight 18.5–24.9 kg/m^2^, overweight 25–29.9 kg/m^2^, and obese ≥30 kg/m^2^.

When assessing cachexia status at cancer diagnosis based on the ICD of 5% weight change within 6 months prior to diagnosis, 608 patients did not have cachexia (69%) and 271 patients were classified as having cachexia (31%; *Table* *1*), similar to our previous findings.^5^, ^13^ In this cohort, stage was not associated with cachexia status (*P* = 0.10). Surgery was the only major therapy modality that was different among cohorts, with 10% more patients without cachexia at diagnosis undergoing resection (63%) than those with cachexia at diagnosis (53%; *P* = 0.005). As expected, patients without cachexia at diagnosis had a higher BMI at diagnosis (29.2 ± 6.3) than patients with cachexia at diagnosis (25.4 ± 5.7 kg/m^2^; *P* < 0.0001). However, there was no association between BMI class and cachexia status at diagnosis (*P* = 0.34). There were also no significant differences in age (*P* = 0.23), sex (*P* = 0.51) or race (*P* = 0.56) between patients without and with cachexia at diagnosis.

**Figure 2:**
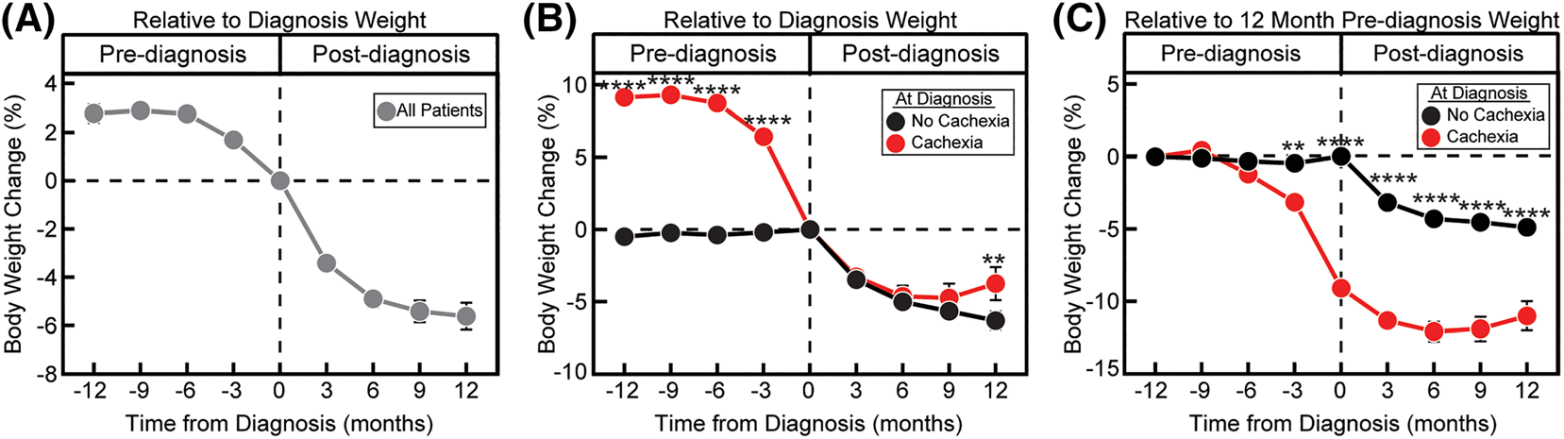
Pre‐ and post‐cancer diagnosis weight change. (A) Overall weight change in all patients. (B) Weight change relative to diagnosis weight in patients without and with cachexia at diagnosis 12 months pre‐ and post‐cancer diagnosis. (C) Weight change relative to initial body weight in patients without and with cachexia at diagnosis 1 year pre‐ and post‐cancer diagnosis. Data are shown as mean ± SEM. ^**^ *P* < 0.01 and ^****^ *P* < 0.0001 based on repeated measures mixed‐effect model using the restricted maximum likelihood method and adjustment for multiple comparisons using Tukey adjustment to compare weight change over time between non‐cachexia and cachexia groups. See *Table* *S1* for patient counts stratified by the indicated time points.

With greater granularity regarding longitudinal weight change pre‐ and post‐cancer diagnosis, we next determined how these GI cancer patients manifested weight loss over time. We attributed weight changes prior to diagnosis as a function of cancer development whereas weight changes post‐diagnosis were related to disease progression and/or treatment side effects. When assessing weight change in the cohort relative to weight at diagnosis, patients on average lost 2.78 ± 0.4% body weight in the 6 months prior to diagnosis (*P* < 0.0001) and 5.4 ± 0.4% body weight in the 9 months after diagnosis (*P* < 0.0001; *Figure* *2* *A*). As seen in *Figure* *1*, 84% of the original cohort of GI cancer patients were excluded from the study cohort due to missing body weight data at diagnosis and pre‐diagnosis. Exclusion of patients without body weight data is necessary but may introduce a selection bias. To account for this selection bias, we compared weight trajectories post‐diagnosis of patients included in the current cohort with those excluded from the cohort. Post‐diagnosis weight trajectories of patients excluded from the cohort (*n* = 2781) did not differ from patients included in the final cohort (*n* = 879; *Figure* *S1*; *P* = 0.2).

When patients were dichotomized by cachexia status at cancer diagnosis based on the ICD, patients without cachexia at diagnosis remained weight stable during the 12 months prior to diagnosis (+0.5 ± 0.5% body weight; *P* = 0.99) and lost 6.3 ± 0.6% body weight (*P* < 0.0001) during the 12 months post‐diagnosis (*Figure* *2* *B*). Patients with cachexia at diagnosis were weight stable between 6 and 12 months prior to cancer diagnosis (+0.4 ± 0.8% body weight; *P* > 0.9999) followed by weight loss of 8.7 ± 0.6% (*P* < 0.0001) within the 6 months pre‐cancer diagnosis. Patients with cachexia at diagnosis continued to lose an additional 4.7 ± 0.7% body weight (*P* < 0.0001) post‐diagnosis. As expected, weight change prior to diagnosis was greater in the patients with cachexia at diagnosis (−9.1 ± 0.8%) compared to patients without cachexia at diagnosis (+0.5 ± 0.5%; *P* < 0.0001). Surprisingly, however, independent of pre‐diagnosis differences in weight change, patients lost similar percentages of weight in the 9 months post‐cancer diagnosis. Patients without cachexia at diagnosis eventually lost 5.7 ± 0.4% body weight post‐diagnosis compared to patients with cachexia at diagnosis whom lost an additional 4.7 ± 0.7% body weight post‐diagnosis (*P* = 0.8; *Figure* *1* *B*). To assess the total body weight changes that occur in GI cancer patients over time, we also evaluated weight change relative to an initial pre‐diagnosis body weight (*Figure* *2* *C*). Patients without cachexia at diagnosis were weight stable pre‐diagnosis and lost a maximum of 4.9 ± 0.4% body weight by 12 months post‐diagnosis. Patients with cachexia at diagnosis had a maximal weight loss of 12.1 ± 0.7% at 6 months post‐diagnosis. Seventy‐five per cent of this weight loss occurred pre‐diagnosis, and 25% of this weight loss occurred post‐diagnosis.

**Figure 3:**
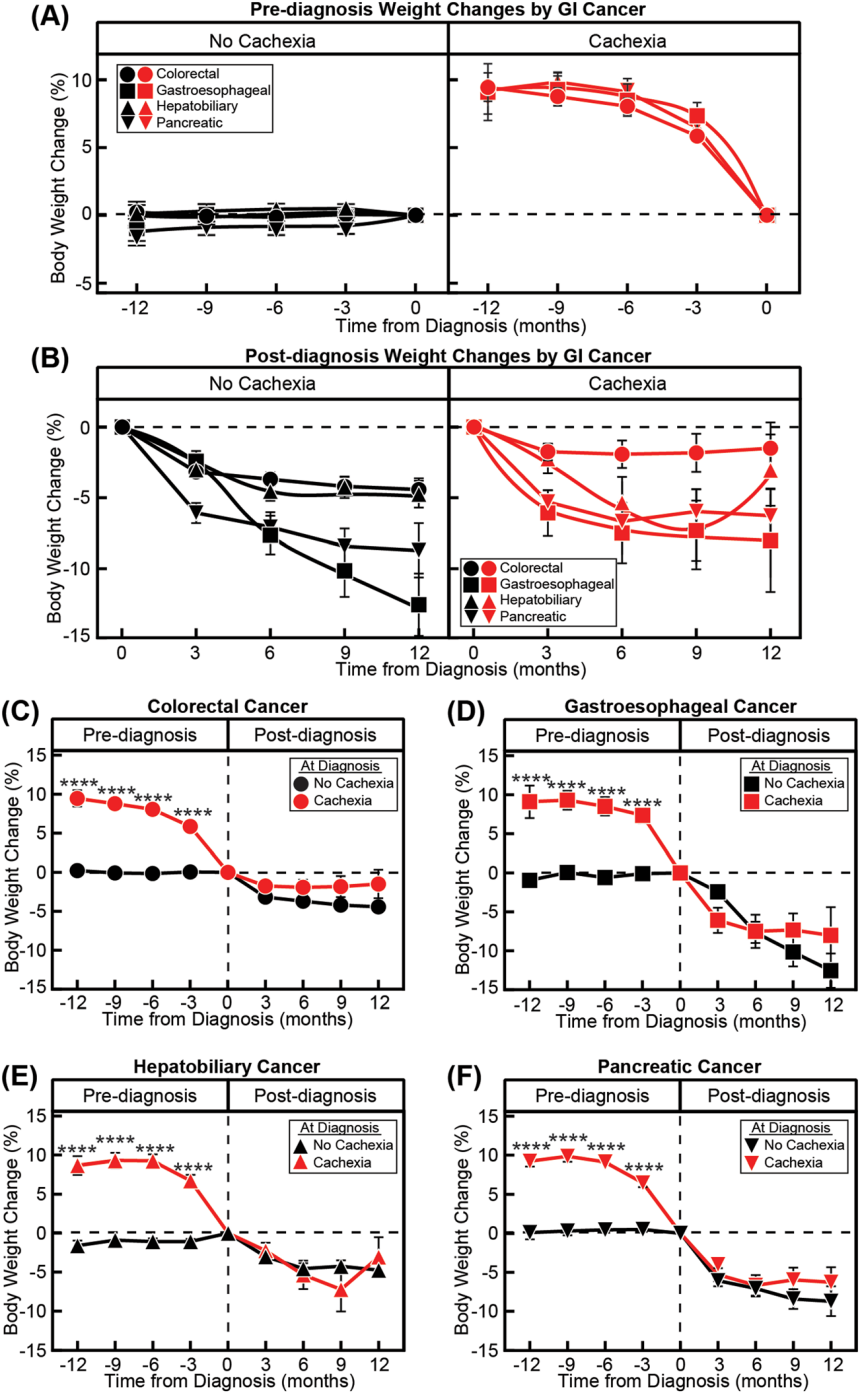
Pre‐ and post‐diagnosis weight change by primary malignancy and cachexia status. Weight change relative to diagnosis weight in patients without and with cachexia at diagnosis 12 months (A) pre‐ and (B) post‐cancer diagnosis by primary malignancy (colorectal, C; gastroesophageal, D; hepatobiliary, E; and pancreatic, F). Data are shown as mean ± SEM. ^****^ *P* < 0.0001 based on repeated measures mixed‐effect model using the restricted maximum likelihood method and adjustment for multiple comparisons using Tukey adjustment to compare weight change over time between non‐cachexia and cachexia groups. See *Table* *S2* for patient counts stratified by the indicated time points.

Because our full cohort consisted of several GI cancers previously associated with cancer cachexia, we next sought to identify any differences in cachexia prevalence pre‐ and post‐diagnosis by primary malignancy. Our cohort was composed of gastroesophageal, colorectal, hepatic, biliary and pancreatic cancers (*Figure* *3*). Among patients without cachexia at diagnosis, weight trajectories pre‐diagnosis did not differ between gastroesophageal, colorectal, hepatobiliary and pancreatic cancer (*P* = 0.4; *Figure* *3* *A*), but were significantly different post‐diagnosis by tumour type (*P* < 0.0001; *Figure* *2* *A*). Similarly, among patients with cachexia at diagnosis, only weight trajectories post‐diagnosis differed as a function of primary malignancy (*P* = 0.8 pre‐diagnosis vs. *P* = 0.0002 post‐diagnosis; *Figure* *3* *B*). In both patients without and with cachexia, colorectal patients lost the least amount of weight post‐diagnosis and gastroesophageal patients lost the most amount of weight post‐diagnosis.

Pre‐diagnosis weight trajectories in patients with colorectal cancer differed between patients without (0.2 ± 0.4% body weight) and with (9.5 ± 0.9% body weight) cachexia at diagnosis (*P* < 0.0001; *Figure* *3* *C*). However, patients without cachexia at diagnosis eventually lost 4.4 ± 0.8% body weight post‐diagnosis (*P* < 0.0001), whereas colorectal cancer patients with cachexia at diagnosis only lost an additional 1.9 ± 0.9% body weight post‐diagnosis (*P* = 0.8). Weight trajectories of patients with gastroesophageal cancers also differed pre‐diagnosis between patients without (+0.9 ± 0.8% body weight) and with (9.1 ± 1.7% body weight) cachexia at diagnosis (*P* = 0.004; *Figure* *3* *D*). The gastroesophageal patients without cachexia at diagnosis eventually lost 12.5 ± 2.2% body weight post‐diagnosis (*P* < 0.0001). Gastroesophageal cancer patients with cachexia at diagnosis lost an additional 7.3 ± 2.1% body weight post‐diagnosis (*P* = 0.02).

Due to the small number of patients with biliary cancer, we combined patients with hepatocellular and biliary cancers into one group, hepatobiliary cancer. Similar to the other primary malignancies, weight trajectories were different pre‐diagnosis between hepatobiliary cancer patients without (+1.6 ± 0.6% body weight; *P* = 0.14) and with (6.7 ± 0.8% body weight; *P* < 0.0001) cachexia at diagnosis (*P* < 0.0001; *Figure* *3* *E*). Evaluation of post‐diagnosis weight trajectories revealed that hepatobiliary cancer patients without and with cachexia at diagnosis lost 4.7 ± 0.8% body weight (*P* < 0.0001) and 7.2 ± 2.5% body weight (*P* = 0.2), respectively (*P* = 0.97). Finally, pancreatic cancer patients without and with cachexia at diagnosis lost 0.1 ± 0.8% body weight (*P* > 0.9999) and 9.2 ± 0.6% body weight (*P* < 0.0001), respectively, prior to diagnosis. Pancreatic cancer patients without cachexia at diagnosis eventually lost 8.7 ± 1.6% body weight during the post‐diagnosis period (*P* < 0.0001). Pancreatic cancer patients with cachexia at diagnosis lost an additional 6.7 ± 1.2% body weight over 6 months post‐diagnosis (*P* < 0.0001).

**Figure 4:**
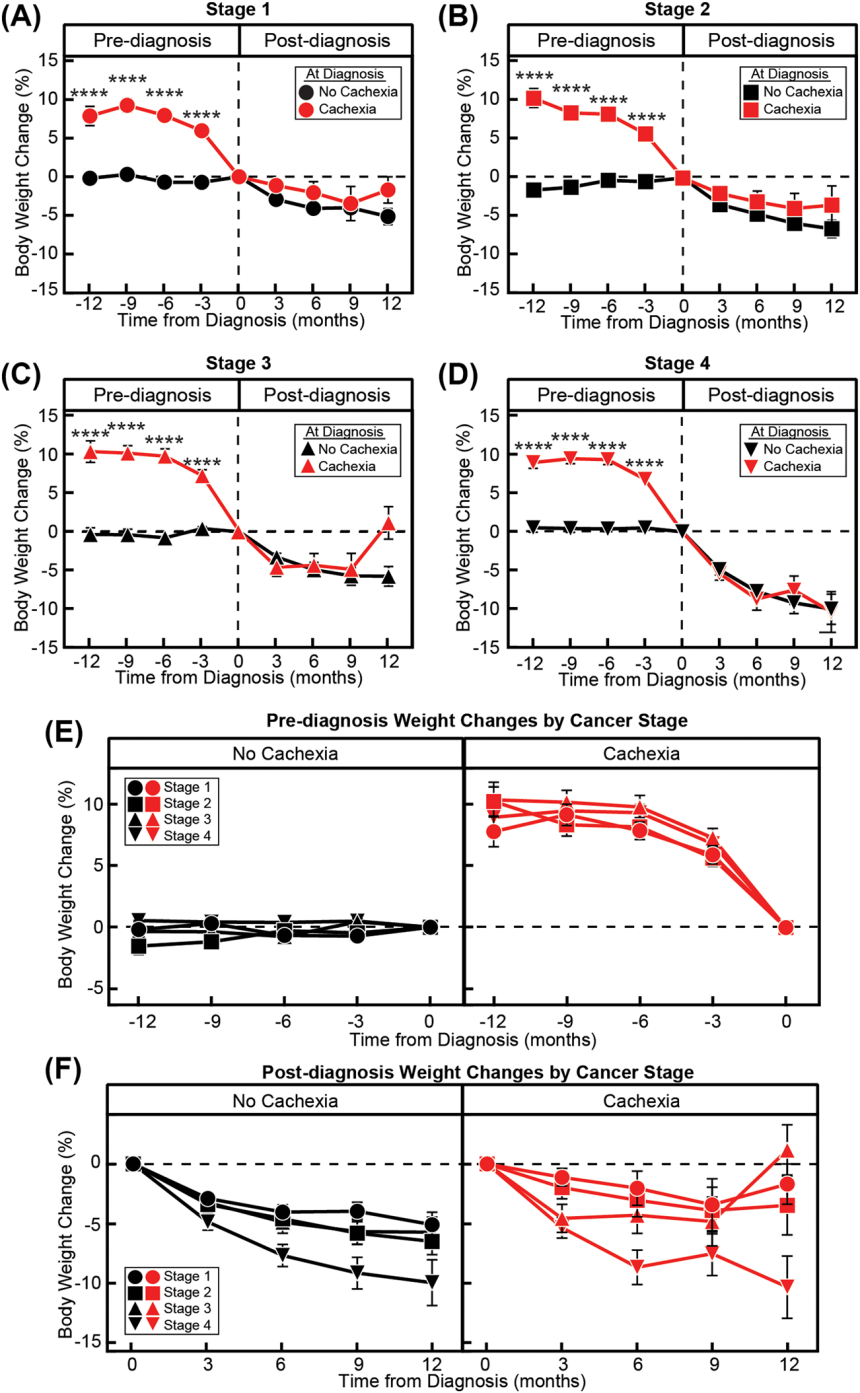
Pre‐ and post‐diagnosis weight change by stage and cachexia status. Weight change relative to diagnosis weight in patients without and with cachexia at diagnosis by stage (Stage 1, A; Stage 2, B; Stage 3, C; and Stage 4, D) 12 months (E) pre‐ and (F) post‐diagnosis. Data are shown as mean ± SEM. ^****^ *P* < 0.0001 based on repeated measures mixed‐effect model using the restricted maximum likelihood method and adjustment for multiple comparisons using Tukey adjustment to compare weight change over time between non‐cachexia and cachexia groups. See *Table* *S3* for patient counts stratified by the indicated time points.

We next investigated whether stage of cancer at diagnosis was associated with alterations in weight trajectories. Within each stage, weight trajectories were significantly different between patients without and with cachexia during the pre‐diagnosis period (all stages *P* < 0.0001), but did not differ during the post‐diagnosis period (all stages *P* > 0.05) (*Figure* *4* *A*–*D*). In patients with cachexia at diagnosis, pre‐diagnosis weight trajectories did not differ between different stages of disease (*P* = 0.11.; *Figure* *4* *E*). However, post‐diagnosis weight trajectories were different between stages (*P* < 0.0001; *Figure* *4* *F*), with Stage 4 disease having the most weight loss during the post‐diagnosis period (10.39 ± 2.2% body weight; *P* = 0.0003). Patients without cachexia at diagnosis showed a similar trend with differences in magnitude of weight loss occurring only during the post‐diagnosis period (*P* < 0.0001) and not the pre‐diagnosis period (*P* > 0.05).

**Figure 5:**
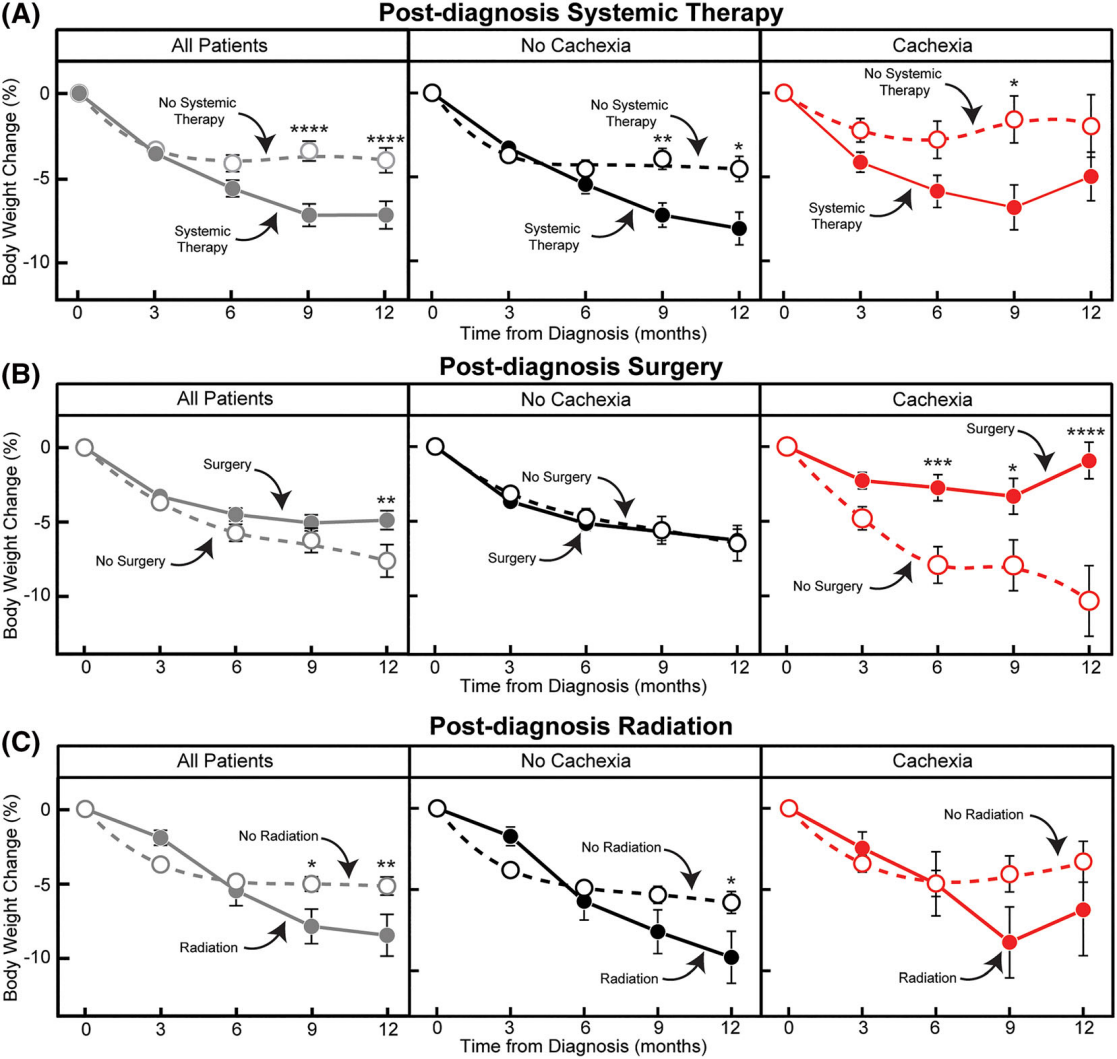
Post‐diagnosis weight change by treatment paradigm and cachexia status. Weight change post‐diagnosis in all patients (grey), patients without cachexia at diagnosis (black) and patients with cachexia at diagnosis (red) by treatment type (systemic treatment, A; surgery, B; and radiation, C). Data are shown as mean ± SEM. ^*^ *P* < 0.05, ^**^ *P* < 0.01 and ^****^ *P* < 0.0001 significance was based on repeated measures mixed‐effect model using the restricted maximum likelihood method and adjustment for multiple comparisons using Tukey adjustment to compare weight change over time between non‐cachexia and cachexia groups. See *Table* *S4* for patient counts stratified by the indicated time points.

Knowing that the different GI primary cancers were treated with a combination of local therapies (surgery and/or radiation) and/or systemic therapy driven by stage of disease, we next assessed how specific treatments influenced weight changes post‐diagnosis in our cohort (*Figure* *5*). Patients who received chemotherapy lost 7.1 ± 0.7% body weight during the post‐diagnosis period compared to those patients who did not receive chemotherapy whom lost 4.1 ± 0.5% body weight (*P* < 0.0001; *Figure* *5* *A*). Cachexia status at diagnosis did not affect this chemotherapy interaction (*P* = 0.27). Overall, patients who did not have surgery lost more weight in the 12 months post‐diagnosis (7.6 ± 1.1% body weight) than those patients who underwent surgery (4.9 ± 0.6% body weight; *P* < 0.0001; *Figure* *5* *B*). This relationship was influenced further by cachexia status at diagnosis. Among patients with cachexia at diagnosis, those patients who did not have surgery lost more weight post‐diagnosis (10.3 ± 2.3% body weight) than those who did have surgery (0.9 ± 1.2% body weight; *P* < 0.0001). Patients who received radiation as a part of their treatment plan lost more weight post‐diagnosis (8.4 ± 1.4% body weight) than those who did not receive radiation (5.1 ± 0.6% body weight; *P* = 0.02; *Figure* *5* *C*). Cachexia status at diagnosis did not affect this radiation interaction (*P* = 0.68). Ultimately, patients without or with cachexia at diagnosis lost weight when treated with systemic therapy or radiation. Patients without cachexia at diagnosis who did not undergo surgical resection lost more weight than those that did after diagnosis. Surgery appeared beneficial for those patients with cachexia at diagnosis in limiting additional post‐diagnosis weight loss.

**Figure 6:**
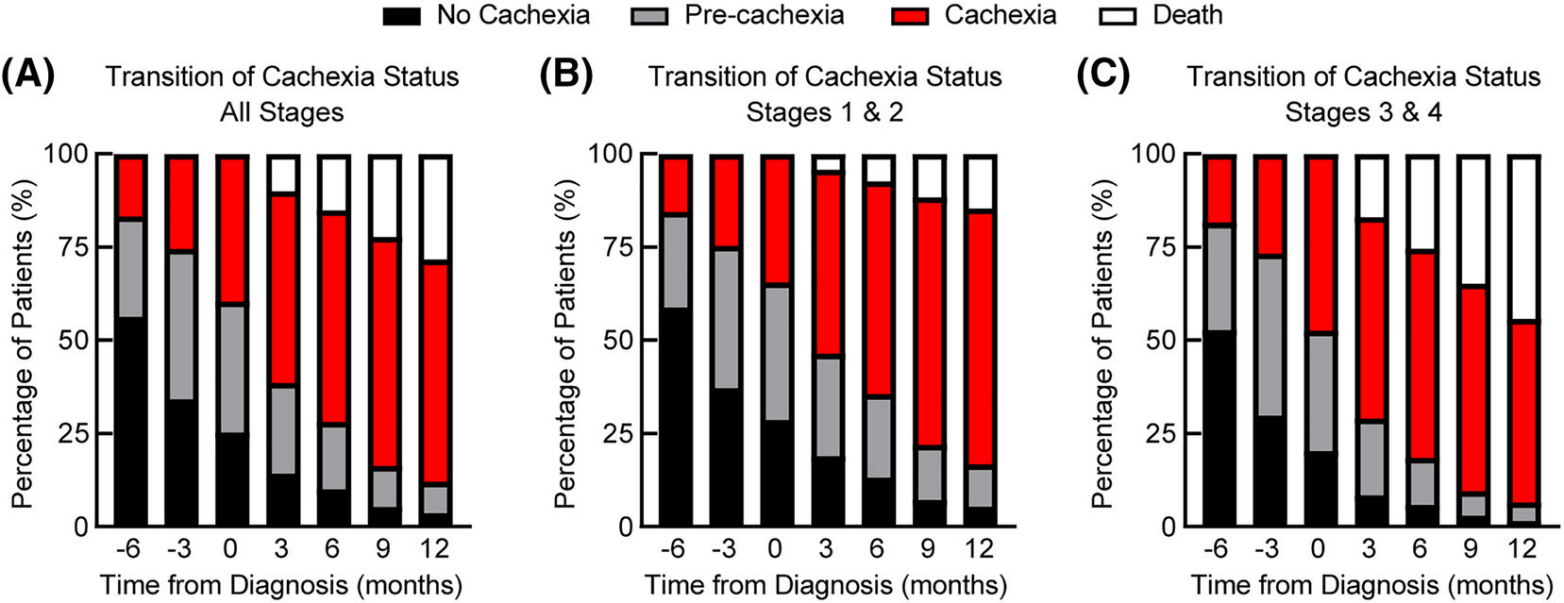
Cachexia transition pre‐ and post‐cancer diagnosis. Patients were categorized as no cachexia (no weight loss or having weight gain in the past 6 months), pre‐cachexia (1–4.9% weight loss if BMI ≥ 20 kg/m^2^ or 1–1.9% weight loss if BMI < 20 kg/m^2^ in the past 6 months), cachexia (≥5% if BMI ≥ 20 kg/m^2^ or ≥2% weight loss if BMI < 20 kg/m^2^ in the past 6 months) or death if the patient had died prior to the time point of interest. Once a patient was considered to have cachexia, they were not categorized as pre‐cachexia or no cachexia at later time points. Transitions were assessed in (A) the whole cohort and in patients with (B) early‐ and (C) late‐stage disease. See *Table* *S5* for patient counts stratified by the indicated time points.

Because the above data demonstrated that patients without cachexia at diagnosis eventually lost significant body weight during the post‐diagnosis period, we next evaluated the transitions of patients from no weight loss (0–1% body weight loss) to pre‐cachexia (1–4.9% body weight loss) and from pre‐cachexia to cachexia (≥5% body weight loss). *Figure* *6* *A* demonstrates how patients transitioned through the stages of cachexia pre‐ and post‐diagnosis. The percentage of surviving patients with no weight loss decreased over time from 56% at 6 months pre‐diagnosis to 5% at 12 months post‐diagnosis. The amount of time patients remained in the no‐weight loss group was 147 ± 5.6 days prior to progressing to pre‐cachexia status. The percentage of surviving patients with pre‐cachexia was maximal at 40% at 3 months pre‐diagnosis and decreased to 12% at 12 months post‐diagnosis. The amount of time patients remained in the pre‐cachexia group was 136 ± 5.5 days prior to progressing to cachexia. The percentage of surviving patients with cachexia increased over time from 17% at 6 months pre‐diagnosis to 83% at 12 months post‐diagnosis. These findings highlight the fact that independent of cachexia status and stage of disease at diagnosis, 95% of patients developed significant weight loss with 83% reaching the ICD of cachexia by 12 months post‐cancer diagnosis. These transitions were significant even if our cohort was separated into Stage 1 and 2 (*Figure* *6* *B*) and Stage 3 and 4 (*Figure* *6* *C*) disease.

## Discussion

One of the most common clinical causes of unintentional weight loss is the development of a GI cancer.^14^, ^15^ Positive associations between weight loss, multiple primary malignancies including gastroesophageal, colorectal, hepatobiliary and pancreatic cancers, and increased morbidity and mortality have been demonstrated.^5^, ^15^, ^16^, ^17^, ^18^, ^19^, ^20^, ^21^ Therefore, monitoring and addressing weight loss in the primary and specialized care settings are of the upmost importance to aid in cancer screening and disease management. However, weight change is one of many clinical variables that can be considered non‐specific to a disease pathology,^22^ and objective longitudinal measures of pre‐diagnosis weight change are not often reported. Furthermore, once a GI cancer diagnosis is rendered, multidisciplinary clinical teams often overlook additional weight changes during the post‐diagnosis period that may further impact morbidity and mortality. A knowledge of the trajectories of weight change for a sustained time both before and after a GI cancer diagnosis would inform us on how weight loss evolves out of cancer development during the pre‐diagnosis and the influence of therapeutic interventions during the post‐diagnosis period.

Using this retrospective cohort in which body weight was measured in the clinic at 3‐month intervals, we were able to quantify body weight trajectories throughout the 12 months pre‐ and post‐cancer diagnosis. Overall, GI cancer patients with cachexia at diagnosis lost ~14% body weight over 12 months pre‐ and post‐diagnosis. Approximately 75% of their weight loss occurred before their cancer diagnosis, and 25% of their weight loss occurred after diagnosis. Meanwhile, GI cancer patients without cachexia at diagnosis had no weight loss pre‐diagnosis but experienced 6.3% weight loss post‐diagnosis. Interestingly, nearly all patients (95%), regardless of the pre‐diagnosis weight loss, eventually developed weight loss with 83% of these patients reaching the ICD of cachexia within 12 months post‐diagnosis. Like pre‐diagnosis weight loss, post‐diagnosis weight loss is also associated with morbidity and mortality in patients with GI cancer,^16^, ^17^, ^18^ and these data suggest the importance of continued weight monitoring with early palliative intervention once patients experience weight loss.

Despite differences in cachexia prevalence, magnitude of pre‐diagnosis weight loss did not differ between stage of disease, primary malignancy or BMI classification at diagnosis. However, patients with GI cancers were weight stable between 6 and 12 months prior to diagnosis and experienced more rapid weight loss in the 3 months prior to diagnosis. Similarly, magnitude of post‐diagnosis weight loss varied significantly between primary malignancies. Patients with gastroesophageal or pancreatic cancer lost more body weight compared to colorectal and hepatobiliary patients during the post‐diagnosis period. These weight trajectory differences may be due to differences in underlying pathophysiology and disease progression of each primary malignancy or due to differences in utilization of antitumour‐directed therapies. Both gastroesophageal and pancreatic cancers can cause structural/obstructive changes, anorexia and metabolic changes, and patients with pancreatic cancer can also have malabsorption due to pancreatic insufficiency all causing severe weight loss.^23^, ^24^, ^25^, ^26^, ^27^, ^28^

In addition to disease pathophysiology, treatment paradigms may affect post‐diagnosis weight trajectories. These effects may be direct such as dysphagia due to radiation therapy or decreased oral intake related to anorexia and odynophagia as side effects of chemotherapy. The treatment paradigm may also be related to stage of disease and curative intent. Surgery is usually included in the care plan for early‐stage disease with curative intent. Although stage was not associated with surgical status in this study, patients with cachexia at cancer diagnosis who did not have surgery are likely to be patients with more advanced disease, and greater weight loss is therefore expected post‐diagnosis. Interestingly, among patients who had surgery, patients without cachexia at diagnosis lost more weight post‐diagnosis than patients with cachexia at diagnosis. Longitudinal weight data in other cohorts are limited; however, preoperative cancer cachexia is associated with an increase in short‐term post‐operative complications.^29^, ^30^, ^31^ More prospective trials are needed to understand the influence of cachexia status and anticancer treatment modality combinations on body weight, metabolic and inflammatory alterations, and clinical outcomes.

Historically, in studies of cancer cachexia patients, a notable absence in our understanding of cachexia progression is the number of patients and lengths of time representing transitions between the different stages of cachexia—no cachexia, pre‐cachexia, cachexia and refractory cachexia leading to death. Our study provides this much‐needed granularity, determining how patients transition through the stages of cachexia. Interestingly, 83% of all surviving patients with a GI cancer in our cohort met the weight loss criteria for cachexia during the 12 months pre‐ and post‐diagnosis. Second, the time in each stage of cachexia in the 12 months post‐diagnosis was quantified. Patients transitioned from no weight loss to pre‐cachexia in about ~4 months and then transition again to cachexia in another ~4 months. These data define critical windows for intervention to attenuate cachexia progression and suggest that the time in each phase of cachexia was not significantly different between patients with early‐ versus late‐stage cancers. Because patient outcomes decline and cachexia becomes less responsive to treatment as it progresses, these time frames identify critical opportunities for intervention prior to cachexia progression. Ultimately, a greater emphasis must be placed on nutritional and palliative care interventions, irrespective of cachexia disposition at cancer diagnosis because nearly all of the no cachexia patients in our cohort eventually developed weight loss within 1 year of diagnosis. Furthermore, a greater understanding of the biology of this disease is necessary to identify potential therapeutic targets because there is currently no Food and Drug Administration (FDA)‐approved medication for this wasting syndrome.

System‐wide and internetwork electronic medical records such as EPIC's Care Everywhere can aid in collation of body weight data from various medical encounters. In addition, patients may self‐monitor their weight at home and provide these data to their physician. However, compliance and accurate data reporting would require patient education focused on weight monitoring as an important indicator. The use of Bluetooth‐enabled devices such as body weight scales and integration of those devices with the EHR would allow for the automated, near real‐time transmission of data to the EHR. Automatically flagging weight loss events in the EHR and alerting healthcare providers may increase the likelihood that these weight change events are addressed and factored into the care plan. Future research should focus on (1) improved methods for monitoring and addressing body weight changes in the clinical setting, (2) the time to referral to a specialist or for a computed tomography (CT) scan and/or endoscope, and (3) other variables that may influence time between a patient engaging with the healthcare system and receiving a cancer diagnosis.

We acknowledge study limitations analogous to all retrospective analysis of EHR data. The data extracted were intended to guide clinical care and not initially collected with the rigour ideal for research. We assumed that the extracted data were accurate, and we screened for potential input errors. We were limited to associative relationships with the ultimate aetiology behind weight change was correlative. Nutrition and pharmacological interventions to attenuate weight loss and preserve nutritional status pre‐ and post‐diagnosis were not considered. Medical nutrition therapy can vary greatly between primary malignancies, anticancer treatments, and patients' weight and nutritional history. Although this is an important consideration, it is best studied in‐depth in a separate manuscript or via a prospective trial. In addition, cachexia is a multifactorial condition in which body weight is a biomarker. In some diseases such as hepatobiliary cancers, comorbid conditions like decompensated cirrhosis resulting in ascites may mask unintentional weight loss.

The findings from this study highlight the importance of monitoring pre‐ and post‐cancer diagnosis weight at every medical encounter whether it be a primary or specialty care, emergency department or inpatient hospital visits. Monitoring body weight can complement established GI cancer screening practices and potentially lead to earlier cancer detection improving patient outcomes. Moreover, our observation that patients underwent significant post‐diagnosis weight loss regardless of cachexia status at the time of cancer diagnosis emphasizes the importance of continual post‐diagnosis weight monitoring for all GI cancer patients. Incorporating practices effective at detecting weight loss throughout cancer disease course can enable physicians to more promptly initiate the necessary palliative and nutritional interventions in GI cancer patient care.

## Conflicts of interest

The authors declared no conflicts of interest. P. I. has worked with AstraZeneca in an advisory capacity and received funding from Incyte unrelated to this work. R. E. I.'s laboratory has received funding from Pfizer Inc. and Incyte unrelated to this work.

## Supporting information


**Table S1.** Patient count for all patients
**Table S2.** Patient count by primary malignancy
**Table S3.** Patient count by stage
**Table S4.** Patient count by therapy
**Table S5.** Patient count for cachexia transition
**Figure S1.** Post‐diagnosis weight change. Weight trajectories of patients excluded from the cohort (*n* = 2,781; *open circles*) did not differ from patients included in the final cohort (*n* = 879; *closed circles*; *p* = 0.2). Data are shown as Mean ± SEM. Significance based on repeated measures mixed‐effect model using the restricted maximum likelihood method and adjustment for multiple comparisons using Tukey adjustment to compare weight change over time between groups.
