# Supplementary material for: A preponderance of gastrointestinal cancer patients transition into cachexia syndrome
Source: J Cachexia Sarcopenia Muscle. 2022 Sep 27;13(6):2920–31. doi: 10.1002/jcsm.13086 (PMC9745477; doi:10.1002/jcsm.13086)
Supplement: Supplementary file 1 — Table S1. Patient count for all patients Table S2. Patient count by primary malignancy Table S3. Patient count by stage Table S4. Patient count by therapy Table S5. Patient count for cachexia transition Figure S1. Post‐diagnosis weight change. Weight trajectories of patients excluded from the cohort (n = 2,781; open circles) did not differ from patients included in the final cohort (n = 879; closed circles; p = 0.2). Data are shown as Mean ± SEM. Significance based on repeated measures mixed‐effect model using the restricted maximum likelihood method and adjustment for multiple comparisons using Tukey adjustment to compare weight change over time between groups. [file JCSM-13-2920-s001.docx]

**Supplemental Tables**

**Supplement Table 1. Patient count for all patients**

| **Time from Diagnosis (month)** | **Total Cohort** | **No Cachexia** | **Cachexia** |
| --- | --- | --- | --- |
| -12 | 385 | 254 | 131 |
| -9 | 473 | 318 | 155 |
| -6 | 528 | 346 | 182 |
| -3 | 714 | 510 | 204 |
| 0 | 879 | 608 | 271 |
| 3 | 763 | 541 | 222 |
| 6 | 663 | 479 | 184 |
| 9 | 590 | 432 | 158 |
| 12 | 518 | 378 | 140 |

**Supplement Table 2. Patient count by primary malignancy**

| **Time from Diagnosis (month)** | **Colorectal** | | **Gastroesophageal** | | **Hepatobiliary** | | **Pancreatic** | |
| --- | --- | --- | --- | --- | --- | --- | --- | --- |
|  | No Cachexia | Cachexia | No Cachexia | Cachexia | No Cachexia | Cachexia | No Cachexia | Cachexia |
| -12 | 93 | 43 | 43 | 13 | 72 | 38 | 46 | 37 |
| -9 | 114 | 52 | 54 | 16 | 94 | 40 | 56 | 47 |
| -6 | 139 | 61 | 56 | 20 | 99 | 47 | 52 | 54 |
| -3 | 199 | 68 | 72 | 25 | 146 | 50 | 93 | 61 |
| 0 | 229 | 88 | 85 | 34 | 187 | 70 | 107 | 79 |
| 3 | 214 | 81 | 75 | 25 | 165 | 59 | 87 | 57 |
| 6 | 194 | 69 | 68 | 20 | 145 | 48 | 72 | 47 |
| 9 | 178 | 64 | 61 | 18 | 130 | 39 | 63 | 37 |
| 12 | 162 | 58 | 57 | 17 | 108 | 33 | 51 | 32 |

**Supplement Table 3. Patient count by stage**

| **Time from Diagnosis (month)** | **Stage 1** | | **Stage 2** | | **Stage 3** | | **Stage 4** | |
| --- | --- | --- | --- | --- | --- | --- | --- | --- |
|  | No Cachexia | Cachexia | No Cachexia | Cachexia | No Cachexia | Cachexia | No Cachexia | Cachexia |
| -12 | 90 | 40 | 65 | 27 | 45 | 19 | 52 | 40 |
| -9 | 124 | 46 | 75 | 35 | 50 | 20 | 68 | 50 |
| -6 | 112 | 50 | 80 | 45 | 53 | 24 | 67 | 59 |
| -3 | 199 | 55 | 107 | 44 | 98 | 33 | 99 | 68 |
| 0 | 227 | 72 | 141 | 59 | 114 | 44 | 118 | 91 |
| 3 | 203 | 63 | 134 | 50 | 106 | 39 | 95 | 66 |
| 6 | 190 | 53 | 121 | 44 | 89 | 35 | 78 | 50 |
| 9 | 171 | 46 | 113 | 40 | 79 | 31 | 68 | 39 |
| 12 | 152 | 42 | 104 | 35 | 72 | 24 | 48 | 36 |

**Supplement Table 4. Patient count by therapy**

| **Time from Diagnosis (month)** | **Systemic Therapy** | | | | **Surgery** | | | | **Radiation** | | | |
| --- | --- | --- | --- | --- | --- | --- | --- | --- | --- | --- | --- | --- |
|  | **No Systemic Therapy** | | **Systemic Therapy** | | **No Surgery** | | **Surgery** | | **No Radiation** | | **Radiation** | |
|  | **No Cachexia** | **Cachexia** | **No Cachexia** | **Cachexia** | **No Cachexia** | **Cachexia** | **No Cachexia** | **Cachexia** | **No Cachexia** | **Cachexia** | **No Cachexia** | **Cachexia** |
| -12 | 128 | 73 | 69 | 40 | 159 | 100 | 38 | 13 | 135 | 73 | 62 | 40 |
| -9 | 184 | 82 | 76 | 45 | 200 | 112 | 60 | 15 | 181 | 81 | 79 | 46 |
| -6 | 181 | 86 | 84 | 44 | 200 | 112 | 65 | 18 | 180 | 85 | 85 | 45 |
| -3 | 268 | 95 | 127 | 57 | 296 | 130 | 99 | 22 | 282 | 104 | 113 | 48 |
| 0 | 318 | 116 | 160 | 67 | 363 | 158 | 115 | 25 | 336 | 119 | 142 | 64 |
| 3 | 286 | 91 | 153 | 65 | 330 | 134 | 109 | 22 | 304 | 100 | 135 | 56 |
| 6 | 234 | 70 | 139 | 50 | 277 | 100 | 96 | 20 | 261 | 75 | 112 | 45 |
| 9 | 200 | 55 | 124 | 42 | 235 | 82 | 89 | 15 | 229 | 63 | 95 | 34 |
| 12 | 175 | 48 | 109 | 33 | 199 | 67 | 85 | 14 | 201 | 53 | 83 | 28 |

**Supplement Table 5: Patient count for cachexia transition**

| **Time from Diagnosis (month)** | **All Stages** | **Stage 1 & 2** | **Stage 3 &4** |
| --- | --- | --- | --- |
| -6 | 305 | 180 | 121 |
| -3 | 382 | 228 | 150 |
| 0 | 528 | 317 | 203 |
| 3 | 685 | 379 | 297 |
| 6 | 780 | 440 | 331 |
| 9 | 738 | 404 | 324 |
| 12 | 694 | 375 | 309 |

**Supplemental Figures**


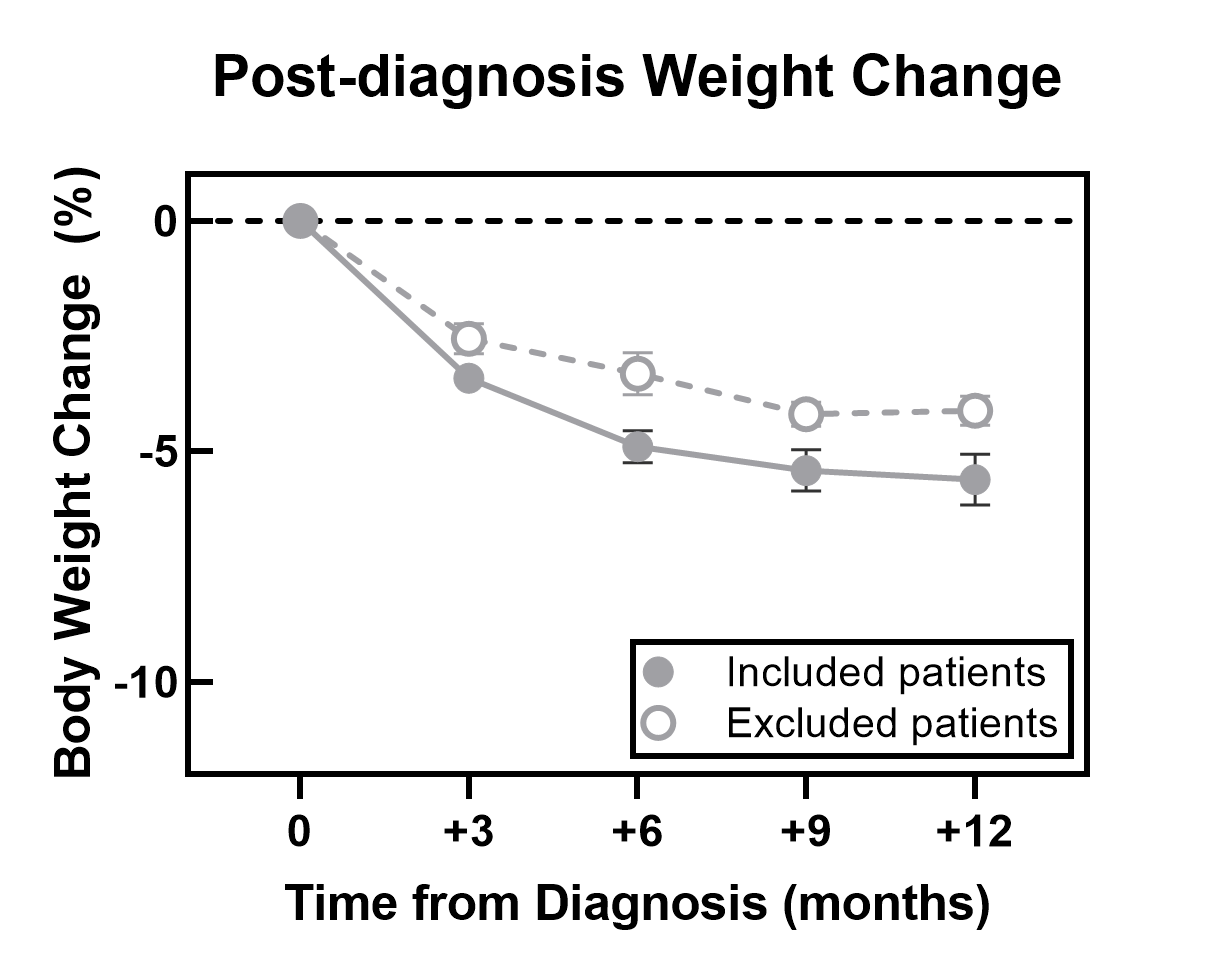


**Supplemental Fig. 1:** Post-diagnosis weight change. Weight trajectories of patients excluded from the cohort (n=2781; *open circles*) did not differ from patients included in the final cohort (n=879; *closed circles*; p=0.2). Data are shown as Mean ± SEM. Significance based on repeated measures mixed-effect model using the restricted maximum likelihood method and adjustment for multiple comparisons using Tukey adjustment to compare weight change over time between groups.
